# Elettra-Sincrotrone Trieste: present and future

**DOI:** 10.1140/epjp/s13360-023-03654-6

**Published:** 2023-01-24

**Authors:** Alfonso Franciosi, Maya Kiskinova

**Affiliations:** Elettra-Sincrotrone Trieste S.C.p.A., Basovizza, 34149 Trieste, Italy

## Abstract

We present an overview of the Elettra-Sincrotrone Trieste research center, which hosts synchrotron and free-electron laser light sources. We review the current status, provide examples of recent achievements in basic and applied research and discuss the upgrade programs of the facility.

## Introduction

Elettra-Sincrotrone Trieste (EST) is an Italian non-profit company of national interest that manages a multidisciplinary research center of excellence based on two light sources, namely the 3rd-generation synchrotron source Elettra since, in operation since 1993 [1], and the seeded free-electron laser (FEL) source FERMI, in operation since 2010 [2]. The light from Elettra feeds 28 and from FERMI 6 beamlines with experimental stations that offer access to state-of-the-art instrumentation for the most advanced spectroscopy, scattering and imaging techniques. The attractiveness of EST has been maintained through constant upgrades of the Elettra and FERMI machines, their beamlines and experimental stations.

The Elettra storage ring has undergone two major upgrades and since 2010 has operated in top-up mode. Elettra currently is the only synchrotron radiation facility routinely operating at two different energies, 2.4 and 2.0 GeV, depending on the users' demand for hard-X-rays vs. VUV photons. It also offers a hybrid mode of operation for time-resolved experiments [3]. Nine out of the 28 beamlines use hard X-rays, eighteen work in the UV, soft and tender X-ray range and one in the IR-THz range. Nine beamlines are operated by the Italian National Research Council (CNR). Several foreign institutions have also established beamlines at Elettra. These are the SAXS beamline of the Graz University of Technology, the Materials Science beamline of the Charles University of Prague. The XRD2 and XPRESS beamlines have been set up and operated in collaboration with the Indian Institute of Science of Bangalore, India. The NanoESCA, SuperESCA and XRF beamlines are operated in partnership with institutions from Germany, Romania and with the International Atomic Energy Agency (IAEA), respectively.

**Fig. 1:**
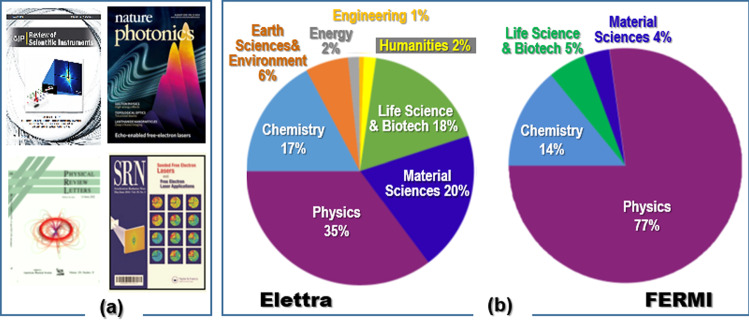
**a** Examples of research achievements at FERMI on the cover pages of journals; **b** research fields of the user proposals that were awarded beamtime at Elettra and FERMI in 2020–2021

FERMI is still the only FEL facility in the world using external seeding to produce fully coherent, intense, tunable and polarized 10–100 fs pulses with unparalleled energy and time jitter stability [4, 5] and allowing generation of two-color FEL pulses [6]. The two FERMI laser lines, denoted as FEL-1 and FEL-2, cover the 100–20 and 20–4 nm wavelength range, respectively. The AIP-RSI cover page in Fig. 1a shows the first results that proved the unique quality of the FERMI source. The intense fully coherent and multicolor pulses with wavelengths shorter than 4 nm, obtained via echo-enabled harmonic generation (EEHG), made the Nature Photonics cover (Fig. 1a) [7]. Scientists of EST and University of Fribourg who demonstrated the generation of attosecond pulse trains at FERMI [8] received the 2020 HZB Innovation Award on Synchrotron Radiation (IASR). The generation of atomic-scale charge current loops that should allow light-induced magnetization at the nanoscale just made the PRL cover [9] (Fig. 1a).

EST also operates a number of ancillary laboratories for sample production and characterization, complementing the experiments at Elettra and FERMI.

About 80% of the hours of beamtime, on average ~ 6400 per year at Elettra and ~ 3600 per year at FERMI, are awarded to international users, based on the ranking of user proposal performed by international peer-review panels. On average annually more than 1200 scientists visit Elettra and FERMI to perform research in a variety of scientific disciplines, as illustrated in Fig. 1b. The results thus obtained in the last five years (2017–2021) are reported in 3364 scientific articles.

More than 50% of the users come from non-Italian institutions, with increasing representations of users from Central-Eastern European and developing countries, because of the ongoing collaborations with of the Central European Initiative (CEI), the IAEA and ICTP.

Since decades, EST is fostering coordinated research activities and know-how transfer through training, schools and workshops that have led to continuous increase in the number of users. EST was an IAEA Collaborating Centre in the period 2005–2014 and was again appointed in 2020 and has ongoing partnership agreements with UNESCO’s International Center of Theoretical physics (ICTP) and many other Italian and foreign institutions [10]. EST is involved in numerous international projects and collaborations [11], including ESS-ERIC, ELI-ERIC, CERIC-ERIC, ERF, Laserlab Europe, EuroFEL and EuPRAXIA, has played an active role in European strategic programs listed in the ESFRI Roadmap and is one of the founders of the League of Accelerator-based Photon Sources (LEAPS). Kyma S.p.A., a spin-off company of EST founded in 2007 to design and manufacture insertion devices, has become a world leader in the field [12].

## Present status and highlights

The versatile beamlines at the Elettra storage ring offer access to almost all synchrotron-based techniques: (i) Photon-in/electron-out spectroscopies (PES-ARPES and XAS), including microscopy, chemical and magnetic imaging; (ii) photon-in/photon-out spectroscopies (XRF, XAS, IUVS-Raman and IR), including X-ray and IR microscopy (XRM and IRM); (iii) X-ray scattering methods (XRD, XRR, SAXS-WAXS). Coherent imaging (CDI and ptychography) is also available at the Nanospectroscopy and TwinMic beamlines. Time-resolved experiments on the ps-ns scale using the hybrid-filling mode of machine operation can be performed at the Nanospectroscopy, SAXS, ALOISA and Gas Phase beamlines and complemented by studies on the fs scale at FERMI.

The six beamlines at FERMI offer a number of time-resolved techniques, including coherent imaging, elastic and inelastic scattering, absorption, emission and transient grating spectroscopies. Helmholtz-Zentrum-Berlin (HZB) bestowed the *Innovation Award on Synchrotron Radiation 2015* on Claudio Masciovecchio for the first demonstration of four-wave mixing (FWM) in the soft X-ray range at FERMI [13]. Four out of six articles in 2016 a special issue of SRN on FELs [14] report results obtained at FERMI, where the study of magnetization dynamics made the cover page (Fig. 1a).

For *operando* experiments, appropriate sample environments have been developed [15–22], the ESCAmicroscopy *operando* set-up featuring on the 2015 ChemCatChem cover [20]. Graphene-sealed cells were demonstrated first at Elettra in the framework of a collaboration with NIST (USA) and TUM (Germany) [21], and more recent advances led to fabrication of graphene encapsulated liquid cells for *in situ* studies of hydrated samples using multiple techniques [22].

Challenging data analysis and management demands are handled by the EST scientific computing group that brings together expertise in high-performance computing, mathematics and physics. Among many achievements, the recently developed dynamic scanning coupled with compressive sensing for faster XRM imaging made the Analyst cover page [23].

Research achievements deriving from the use of EST facilities are abundant, and a selected number of them are summarized in the Elettra Highlights and Top Stories [24]. We will mention only a few examples in what follows.

Since its start, EST has been very active in the application of photon-in/electron-out spectroscopies, now used at 13 beamlines. They include high-resolution PES, angular-resolved PES (ARPES), time-resolved PES (TR_PES), scanning photoelectron microscopy (SPEM) and X-ray photoemission microscopy (XPEEM). Thanks to the synchrotron light, these techniques have become very powerful and in high demand for studying quantum materials, 2D materials, nanostructures and for exploring surface and interface phenomena related to catalysis, energy and electronic devices, chemical sensing and biomedical applications. In particular, the response to operating conditions and external stimuli—temperature, radiation, electric and magnetic fields—are main targets for understanding the functionality of such systems.

**Fig. 2:**
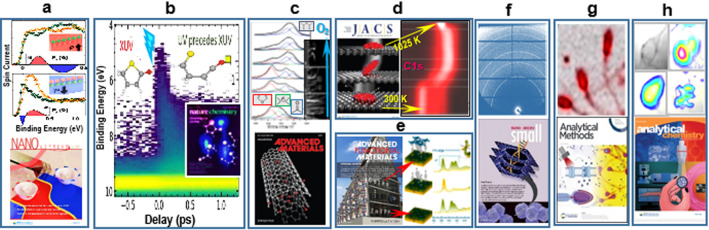
**a** Ferroelectric control of the spin texture in GeTe, verified by spin-polarized spectra and spin asymmetries (P_x_) of Te (top)- and Ge (bottom)-terminated surfaces [26]; **b** photoelectron yield as a function of binding energy and time delay after the UV pump pulse, illustrating the depopulation of the ground state (yellow) and population of excited states including ring opening [31]; **c** CNTs oxidation where the presence of defects encoded in the C 1 s spectrum of each CNT account for different consumption rate and type and abundance of the formed oxygenated functional group [32]; **d** molecular lifting, twisting and curling, encoded in C 1 s spectra during temperature-induced coronene dehydrogenation [34]; **e** coherent layered film grown on an archetypal Fe electrode—the N 1 s XAFS spectra characterize each construction steps during self-assembly and lateral ordering of the molecules [38]; **f** SAXS of bio-mimetically mineralized composites as delivery systems for gene therapy [58]; **g** superimposed Na XRF and absorption XRM maps of sperm cells [62]; (h) visible and IRM images of lipids, proteins and carboxyl groups in live cells [63]

Results of spin-resolved ARPES studies of quantum materials, attractive candidates for the realization of spin-based non-volatile transistors, performed at the APE beamline of Elettra made the Nature Materials cover [25]. Based on the uncovered correlation between ferroelectricity and spin texture and inversion in GeTe, crafting the spin texture via ferroelectric patterning for devices with engineered spin configurations made the NanoLetters cover (Fig. 2a) [26].

The momentum microscope with imaging spin filter at NanoESCA has allowed researchers to measure simultaneously the spin polarization of photoelectrons in the whole surface Brillouin zone. Results for cobalt showed that in itinerant ferromagnets electron correlations are of nonlocal origin [27]. *Operando* studies of memristive devices with XPEEM at NanoSpectroscopy shed light on the formation mechanism, chemical and electronic structure of so-called conductive nano-filaments due to oxygen vacancies created in SrTiO_3–*x*_ and Ta/Ta_2_O_5_/graphene devices [28, 29].

After pioneering *operando* experiments with electronic devices, where the single 2D material channel is only a few µm^2^ in size, the NanoARPES at Spectromicroscopy is endorsed as an attractive tool for understanding functionality of wide range 2D devices and other heterostructures. For the first studied ‘real’ devices, consisting of graphene on monolayers of MoS_2_ or WSe_2_ atop of a BN dielectric, the shifts of semiconductor bands relative to those in the graphene when applying a gate voltage indicated that these two materials do not hybridize [30].

Understanding ring-opening dynamics of heterocyclic molecules is crucial for using such functional ingredients for molecular switches in electronic devices and light-driven bio-reactions. Thanks to the full coherence of FERMI FEL-1 and FEL-2, photo-induced progressive C-S separation of thiophenone and further evolution to open and close ring states was visualized with resonant PES at the LDM beamline. Such snapshots of on-going photoreactions can guide technology processes, and the results made the Nature Chemistry cover (Fig. 2b) [31].

PES microscopy and imaging available at four Elettra beamlines have opened new prospects for exploring individual nano-materials. The finding that the density and type of defects, encoded in the C1s spectra of each carbon nanotube (CNT), account for the different consumption rate upon oxidation, witnessed in the C 1 s images and O 1 s spectra, made the Advanced Materials cover [32] (Fig. 2c).

Some of the most studied functional systems at Elettra are those relevant to catalysis, which is at the core of the chemical industry, fuel and energy production, conversion and storage, as well as in sensors. The first ‘fast’ PES studies of surface reactions started at SuperESCA in 1994 [33]. As illustrated in Fig. 2d, the conformational changes of coronene during temperature-induced dehydrogenation on Ir(111) surface, evidenced by the evolution of the C 1 s spectra, deserved a JACS cover page [34]. An AngewChem cover advertised a PES study of Pt-CeO_2_ catalysts at the Materials Science beamline, reporting a route to reduce the use of Pt in fuel cells [35]. In this field, the use of the FERMI to explore ultrafast dynamics allowed observation of ultrafast transfer of electrons from Ag to CeO_2_ in semiconductor/plasmonic photocatalyst, excited by visible light [36].

Chemical and microstructural evolution of electrodes and electrolytes during operation of electrochemical devices, shedding light on their performance and aging have been explored at many of the Elettra beamlines. The first ‘microscopy’ results, obtained a decade ago, made a central picture on the Chemistry European Journal cover [37]. The results of studies at the ALOISA beamline of molecule/electrode contact, obtained via growth of 2D + n heterorganic film on Fe electrode, which ensures ultra-fast charge transfer and device miniaturization, made the Advanced Functional Materials cover (Fig. 1e) [38]. Extensive structural studies regarding aging of energy devices are ongoing at SAXS, MCX and XAFS beamlines [39]. Very recent X-ray tomography studies, exploiting dendrite suppression during cycling of battery anodes, made ChemElectroChem cover [40]. Elettra scientists were Guest Editors of special issues of the Journal of Physics D [41] and Applied Sciences [42], dedicated to synchrotron-based methods for battery studies, where 4 out of 15 articles report results obtained at Elettra beamlines.

The versatility of XMCD and XMLD has allowed studies of the response of magnetic nanostructures to external stimuli, which is the key for advancing the design of new electronic devices. One of the first observations of reversible changes in skyrmion size under the application of a magnetic field was made by XPEEM at the Nanospectroscopy beamline of Elettra [43]. As noted above, the multiple polarized and fully coherent pulses produced by the FERMI FEL-1 and FEL-2 lasers have instantly attracted the attention of the international community as a tool for exploring ultrafast magnetization dynamics [14, 44, 45] with direct access to complex multicomponent materials using the two-color FEL mode [46]. Magnetic dynamics is the topic of 17 out of 45 publications produced by the DiProI beamline of FERMI in 2016–2021 [47]. Detailed studies of ‘self-induced’ demagnetization in single-shot experiments provided important guideline for the selection of FEL pulse fluence and duration [48]. The recent first experimental proof of magnetic helicoidal dichroism is paving the road toward the use of structured light spectroscopies for exploring chiral systems [49]. Transient grating (TG) experiments with specially designed electromagnet at the EIS-Timer beamline of FERMI provided the unique capability to study magnetic dynamics at the nanoscale by creating transient magnetization gratings with variable periods [50].

Design and fabrication of many functional materials have been improved by the ability to follow microstructural changes when varying the chemical environment, temperature and pressure. Bioinspired films are vital cradles, and the results of *in situ* GISAXS studies of the structural evolution of films from the cephalopod protein reflectin during self-assembly made the Advanced Materials cover [51]. Fabrication of dye-free hybrid solar cells with enhanced light harvesting, based on nano-imprinted mesoporous titanium films verified by GISAXS, was illustrated on an Advanced Functional Materials cover [52].

Metal organic frameworks (MOF), promising ultra-porous materials for a variety of applications in catalysis, batteries, sensing, gas storage, micro-electronics, drug delivery, etc., have extensively been studied at the SAXS and XPRESS beamlines of Elettra. Recently, new structural insights of MOFs under combined mechanical and gas adsorption stimuli were obtained at the XPRESS [53]. Nanoscale MOF patterning, a crucial step toward micro-device fabrication, recently performed at the DXRL beamline and verified by GISAXS deserved a Nature Materials cover [54].

In the field of life sciences, along with protein crystallography, extended research is ongoing using imaging, SAXS, XRM and IRM. At Elettra, we started two decades ago a clinical program based on X-ray phase contrast imaging [55]. Dedicated initially to mammography, the SYRMEP beamline is now a cradle of multiscale biomedical imaging, bridging the gap between preclinical research and patient applications [56].

Numerous SAXS studies are relevant to biomedicine and pharmacology, and medical devices for SAXS biological studies are fabricated at the DXRL beamline of Elettra [57]. Among the SAXS outcomes is the DNA@MOF biocomposite validated as intracellular gene delivery vehicle, which made the SMALL cover page (Fig. 2f) [58] and the efficient encapsulation and transport of partially fluorinated drug molecules, which made the AngewChem cover [59].

XRM at TwinMic [60] and IRM at bio-SISSI [61] are providing complementary morphologic, chemical and biochemical information easing the diagnostics and therapies screening. The recent XRM studies of ovary tissue and sperm cells were recognized by an Analytical Methods cover page for their impact on reproductive medicine (Fig. 2g) [62]. Numerous XRM investigations of tissues and cells for assessing the health hazards of nanoparticles and nano-fibred pollutants are shedding light on toxicity mechanisms [60]. The advantage of IRM for live cell studies was used for monitoring *in situ* biomolecular processes in cancer cells in a recent study that made the Analytical Chemistry cover page (Fig. 2h) [63]. IRM also has revealed the response of different biomolecules relevant to the cellular damage caused by X-ray irradiation. The EIS-TIMEX beamline at FERMI was used to determine the low-frequency vibrational modes in ibuprofen molecules, introducing and new method to determine the biological activity of pharmacological molecules. In the framework of the EU-EXSCALATE4COV project, a method to predict more precisely which molecules can inhibit the main SARS-CoV-2 protease was developed [64].

Environment-relevant studies are also a hot topic, exploring the fate of the contaminations created by human actions and accumulated in soils, plants, oceans and air using complementary XAFS, XRF, IRM, XRM and imaging techniques. Numerous studies at XRF and TwinMic beamlines have revealed the presence toxic species in plants, aerosols and particulates in ambient air and most recently also the uptake of engineered nanomaterials in plant tissues [65]. Another representative recent example is the incorporation of microplastics in marine organisms [66] detected by IRM.

Cultural heritage studies, spanning over archelogy, paleontology, restoration, human evolution and history, are an important topic at EST, reinforced by the fact that Italy is hosting an unparalleled number of UNESCO world heritage sites. Increasingly desired are synchrotron-based nanoprobes, especially for the characterization of tiny and complex micro-samples, which are typical samples that can be extracted from archaeological and artistic artifacts. A report of the achievements in cultural heritage studies performed at TwinMic, XRF, XAFS, MCH, SYRMEP, ESCAMicroscopy and SISSI beamlines and in collaboration with ICTP was recently published in the Handbook of Cultural Heritage Analysis [67].

## Elettra 2.0 project and FERMI 2.0 upgrade plan

**Fig. 3:**
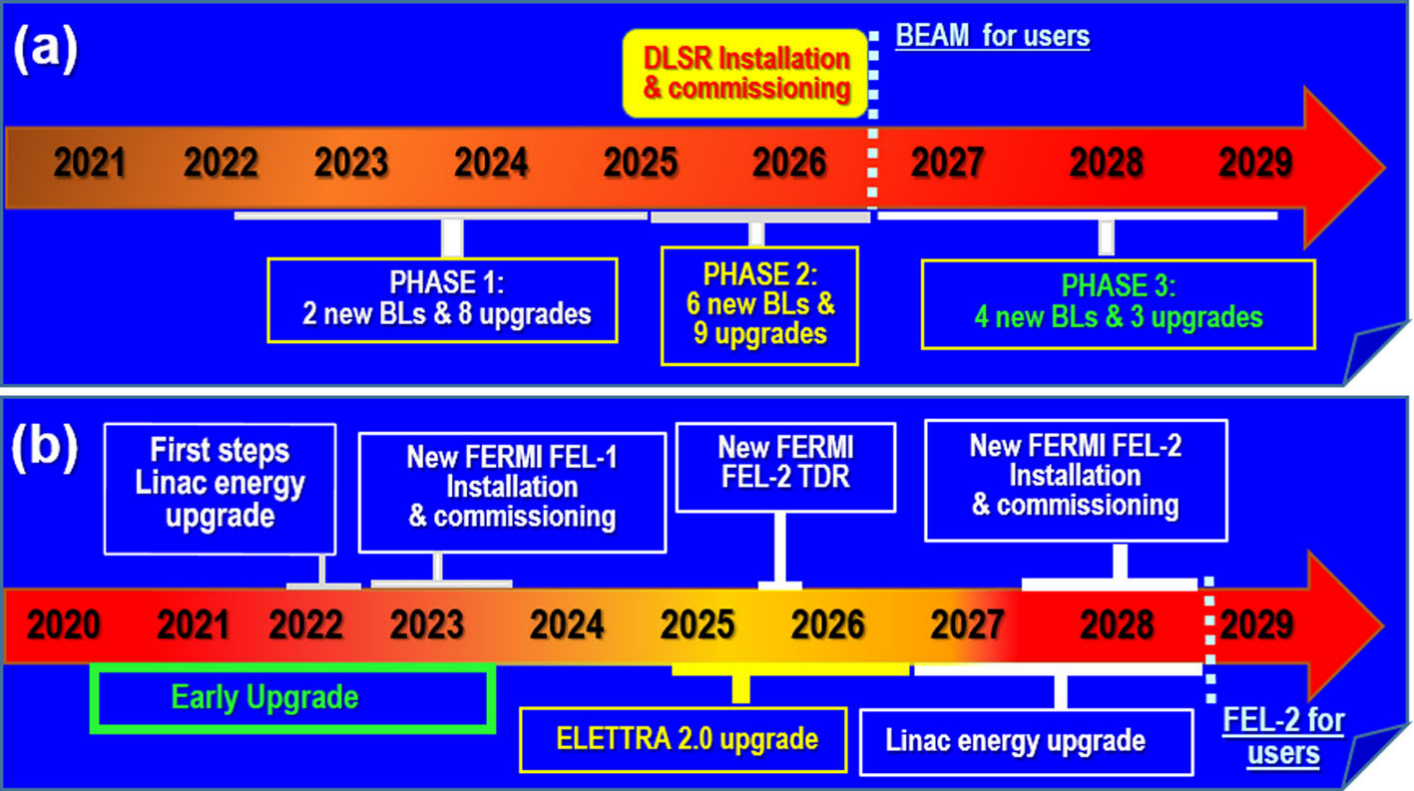
**a** Time line Elettra 2.0 Project [68]; **b** Time line Fermi development [69]

After nearly 30 years of service to the international research community, the Elettra synchrotron radiation source will be replaced by the new Elettra 2.0 4^th^ generation light source [68]. The Elettra 2.0 storage ring will employ a symmetric six-bend enhanced (S6B-E) achromat lattice and will operate predominantly at 2.4 GeV, responding to the increasing user demand for harder X-rays. The brightness will increase by ~ 35 at 1 keV and by ~ 160 at 10 keV. The coherent fraction is expected ~ 30% and ~ 3% at 1 and 10 keV, respectively. These parameters of Elettra 2.0 will boost the spatial, energy and temporal resolution of all imaging, scattering and spectroscopic techniques allowing new methodologies for *in situ* and *operando* characterization of all types of functional matter. A distinct additional feature under consideration is the implementation of deflecting cavities to provide pulses in the few ps range with sufficient intensity and high repetition rate. Such short pulses, combined with tunability, polarization control and pump-probe capabilities, will be opening new research opportunities in the time domain for the study of the transient phenomena that govern functionality in many complex physical, chemical and biological systems. The project is ongoing and, as illustrated in Fig. 3a, the new storage ring and the first beamlines should start operation in 2026.

Elettra 2.0 will have up to 32 beamlines, 20 of the present ones should be upgraded, and 12 new are scheduled to be built. Eight of the new ones are hard X-ray beamlines, using superbends or insertion devices. The planned beamlines at Elettra 2.0 will include a balanced representation of the synchrotron-based methods, considering all research fields and the grand social challenges, as described in detail in the Science Drivers section of the TDR [68]. Some of the planned new beamlines are outlined below.

Phase 1 superbend (4–60 keV) beamline SYRMEP–Life Science for multiscale high resolution tomography replaces the present SYRMEP. It includes clinical applications as well, where the coherence-dependent phase contrast imaging will have a strong impact in studies of organic and bio-matter. Phase 2 superbend XAS-SB will reach the K-edges of more elements, gaining in spectral and time resolution as well. Phase 3 superbend XPRESS will be dedicated to studies of matter under extreme conditions. Other new hard-X-ray beamlines using in-vacuum undulators will reach 15 keV with microprobe capabilities: µXRD and µXRF will replace the present ones and a new High Brightness SAXS (HB-SAXS) will offer very high spatial resolution. The HB-SAXS will also gain from the high coherence, where XPCS, both in normal and grazing incidence scattering, will allow for cutting-edge studies of processes that control the evolution of complex nanostructured matter, spanning over biology, chemistry and operating electronic and energy devices.

A new tender X-ray (0.5–6.5 keV) elliptical undulator beamline, called APE-TX, complementing hard X-ray PES (HXPES) with XAFS for exploiting matter properties with variable probing depth, will add the missing information for interfacial phenomena controlling the functionality of devices, the exotic properties of quantum systems and other technological materials. The new MOST beamline (10–2000 eV) exploiting two elliptical undulators is devoted to gas phase and chemistry studies. It will replace the existing CIPO and GasPhase beamlines, offering our users all photon-in/photon-out and phonon-in/electron-out spectroscopies. A new elliptical undulator (450–3500 eV) CDI beamline, developed in collaboration with NSLS-II, will use all CDI approaches for exploring with variable depth sensitivity structural defects and the evolution of the magnetic, electronic and chemical structure of a wide range of quantum and complex nanomaterials and interfaces.

Two main upgrades envisaged for FERMI 2.0 are described in details in the CDR document [69]. In the first phase, we will implement the EEHG operating scheme for the new FERMI FEL-1 laser line, pushing the minimum wavelength down to 10 nm. In the second stage, to start in 2027, a substantial increase in the LINAC energy, an upgrade of the undulators and a conversion from the HGHG operating scheme to an EEHG first stage, followed by an HGHG second stage, will allow the FERMI FEL-2 laser line to reach photon energies well beyond the oxygen K edge and pulse widths down to a few fs. The first upgrade steps of the LINAC are planned to start end of 2022, the TDR should be completed in 2025, and the plan, as illustrated in Fig. 3(b), is to open a new FERMI FEL-2 laser line to users end of 2028.

## Next scientific challenges and concluding remarks

The notable advances in multi-scale characterization of all types of matter, described in the previous sections, will lead to new scientific discoveries. They will speed the design and production of all types of materials and devices and will deepen our understanding of how the bio-nature functions and responds to external challenges. This, in turn, will lead to breakthroughs in our response to major societal challenges relevant to technology, energy, environment and health. Below are only a few examples from ref. [68].

The great challenge for accelerating the advances in electronic and processing devices based on quantum materials is to understand the specific role of the interactions between device constituents in device performance. Multimodal analysis using nano-ARPES with spin-detection, XPEEM-XAFS combined with XPCS, HXPES-XAFS, CDI and SAXS-XPCS will allow us to explore the electronic, magnetic and chemical structure of the constituents in the coexisting phases and at the interfaces down at least to the 10–20-nm scale, as well as the fluctuation dynamics in response to applied fields and temperature changes on the ps time scale.

It should be noted that nano-IR could also be used for probing the presence of surface plasmons in nanostructured quantum materials and investigating the behavior of plasmonic materials in a strong electric field. The optical sub-ps switching dynamics on the nm scale in selected devices will be further explored at FERMI with the advanced scattering and multidimensional spectroscopy methods.

Vital target of energy and environment-relevant research are functional materials and interfaces present in all types of energy devices and catalytic systems. The complexity of these systems is multilevel and controlled by diverse multiscale phenomena occurring on very wide time scales—from fs for bond breaking and bond formation to seconds and minutes for the mass transport processes. Elettra 2.0 beamlines will offer more advanced *operando* capabilities combining complementary soft, tender and hard X-rays techniques with the desired spatial, spectral and depth resolution. Soft X-ray AP-SPEM with surface sensitivity, tender XRM with nano-XAFS, nano-XRF and resonant ptychography, hard X-ray XCT, µXRD, SAXS and XPCS will all be made available at Elettra 2.0. Important contributions are expected to better understand the effect of size-dependent properties on the catalyst performance, in particular in the case of electrochemical and photocatalysis reactions.

The observed magnetic field effect on catalytic reactions has revealed the critical role of spin and localized quantum interactions, which can be verified by ARPES and XAFS using XMCD and XMLD. Further on, the ps time resolution and MHz repetition rate of Elettra 2.0 will be opening unique opportunities to study charge transfer processes, in particular in photocatalysis relevant to the carbon–neutral energy economy. Pump-probe techniques using a light pulse to trigger the reaction and probing with transient PES and XAS the intermediate reaction states are under development and will become routine thanks to the beam parameters of Elettra 2.0 with the option to push time resolution to the limit at FERMI.

Crucial for solving grand challenges in the health field for humanity and biological organisms in general is understanding the functionality of biomatter, where the organization on the nanoscale plays a dominant role in intercellular interactions. For addressing these issues, we will go beyond macromolecular crystallography thanks to the new opportunities opened by Elettra 2.0. Using the *in situ* X-ray imaging (XRM-ptychography and XCT), scattering (µXRD, HB-SAXS & XPCS) and micro-spectroscopy (µXRF, µXAFS, IRM) details in the organization and bio-reaction cycles of multi-component macromolecular structures will be obtained on the scale of sub-organelle and cell dimensions. Such findings will be the key to curing many diseases, deactivating viruses and unraveling complex environment problems.

Last but not least, the use of artificial intelligence and machine learning and adding advanced automation of experiments and data storage and analysis will allow us to greatly expand the *in situ* and high-throughput capabilities of Elettra and FERMI beamlines. The ongoing efforts also aim at coupling the advanced analytical tools with appropriate modeling and high-performance computing for a more complete interpretation of the experimental data and proper selection of the next targets.

To address the grand scientific and social challenges and serve the international user community, collaboration with external institutions and partners is of fundamental importance for our facility. Creating new networks and attracting more researchers from industry to close the gap between basic and applied research are the vital strategic targets, which, in turn, will foster instrumental innovation and new application concepts.
